# Might the Mothers of Premature Babies Feed Them and Devote Some Milk to the Milk Bank?

**DOI:** 10.1155/2018/3628952

**Published:** 2018-12-02

**Authors:** Pasqua Anna Quitadamo, Giuseppina Palumbo, Liliana Cianti, Matteo Luigi Napolitano, Ciro Coviello, Paola Lurdo, Massimiliano Copetti, Maria Assunta Gentile, Pierpaolo Cristalli

**Affiliations:** BLUD, Fondazione IRCCS Casa Sollievo della Sofferenza, UO TIN-Neonatologia, San Giovanni Rotondo, Italy

## Abstract

The breast milk is the gold standard food for the feeding of the premature baby: it is the natural way to provide excellent nutritional, immunological, and biological nutriment so as to facilitate a healthy growth and the development of the infants. When the breast milk is not available, the alternative is represented by the donated milk. The mothers of premature infants are important opportunity if we consider the fact that they could devote some milk both because they provide a food which is closer to the needs of the vulnerable category of newborns and because it is, for the mothers, a way to overcome the detachment and the psychological trauma of a premature birth. There are no data on this kind of donation. The aim of the study is to evaluate the contribution of the milk donation to the HMB of CSS by women who gave birth to premature infants of gestational age <35 weeks and to analyze the macronutrient composition of the “preterm” donated milk. The CSS HMB has recruited 659 donors totalling 2236 liters of donated milk over a period of 7 years. 38 donors (5.7%) gave birth to a gestational age <35 weeks. Almost 20% of the donated milk comes from mothers of premature babies and this is a very important fact because it shows the huge potential belonging to this category of mothers. Taking into account the parameter regarding the birth weight, it was found that VLBW mothers contributed for 56% to preterm donation while ELBW mothers contributed for 41%. By evaluating the variable gestational age, about 40% of the average total donation derives from mothers who gave birth before the 25 weeks, while a contribution of 46% is attributable to the category of newborns with a GA between 25 and 32 weeks. Besides, some other exceptional examples can be outlined. Regarding the correlation analysis DM resulted in negative correlation with GA weeks (r=-0.31, p=0.058) and with BW g (r=-0.30, p=0.068) achieving values which are very close to the significance. The comparison between the donor volume averages of the preterm and full-term groups is statistically significant. The composition data are in line with the literature: there is an increase by 18 % in the protein component of the milk deriving from the mothers of the premature infants; the gap in carbohydrates is less significant (5-6%) and the gap in calories is similarly low being only 2% higher than the single donor milk and 11% more than the pooled milk. The data on the lipids line up to single donor term milk, while it grows by 24% compared to the pooled one. The study shows that even at very low or extreme gestational age it is possible to obtain an appropriate production of breast milk. This awareness becomes a fundamental starting point for the activation in a standardized way of all the strategies of promotion and support of food that have proven effective with the HM in NICU.

## 1. Introduction

The breast milk is considered the gold standard food for the feeding of premature babies. The recent discoveries [1–8] on the inimitable properties of the breast milk—a sophisticated network of thousands of bioactive factors with innumerable functions, not fully known yet, especially of defense against infections, with a marked biodiversity and a high cellularity, as well as a specific nutritional value due to the particular nature of the nutrients-make it an irreplaceable dynamic fluid also for the effects outlined during the infancy, the adolescence, and the adulthood [9–17].

The breast milk is defined a health elixir in the short and long terms for the premature infant and is considered an “essential drug” in the NICU. The milk produced by the mothers of premature babies has specific characteristics of composition, suitable for a preterm baby that grows rapidly and is still vulnerable.

The protective effect of the breast milk on the NEC [18–25] is an accepted fact; it is also widely demonstrated that the extended use of the breast milk in feeding the premature babies, especially the VLBW, significantly reduces the incidence of all complications of prematurity [19, 26, 27]. For this reason, the scientific societies and the organizations that deal with health, indicate the mother's milk as the first choice; otherwise, whether the mother's milk was not available, one should refer to the donated milk. Although the evidences on the protective effect of the donated milk on the pathologies related to the premature birth are not so overwhelming, the only clearly demonstrated evidence is on the NEC, the data available, and the experience of the centers that dispose milk banks evoking the conservation of the majority of the beneficial effects of human milk [27–35], within the framework of the treatment methods of the human milk. Furthermore, the presence in NICU of a HMB acts as a facilitator for the nursing of term and preterm infants [36].

We do not know the amount of milk referring to each mother (there is a lack of data) and how many women, who have given birth prematurely, succeed at feeding their children exclusively with their precious milk. What is more unknown is the frame of reality regarding the donations to the milk banks.

There are no articles evaluating the amount of human milk devoted by women who gave birth prematurely nor any reports of sporadic cases of women who succeeded at feeding their premature babies exclusively with their own milk devoting a part of their milk to a milk bank.

## 2. Description of the Study

### 2.1. Aims

1. Contribution's evaluation of the milk devoted to the HMB of CSS by women who gave birth to premature babies.
2. Analysis of the macronutrient composition of the donated milk.

## 3. Materials and Methods

The data analyzed in this paper concern the HMB of CSS, active since 2010, recruiting mothers of children born in the aforementioned center.

The study includes mothers of premature infants of gestational age under 35 weeks who gave birth in our center from 2010 to 2017 and who donated part of their milk to the HMB.

A distribution for categories of the volume devoted to the milk bank was performed with regard to the gestational age and to the birth weight.

Our source is a database containing information about: donors, milk (donated), and newborns (fed).

## 4. Statistical Methods

Subjects' clinical characteristics were reported as mean (± standard deviation) and as frequencies and percentages for continuous and categorical variables, respectively, overall and according to gestational age (22-25 w, 26-32 w, and 33-35 w), and birth weight (<1000 gr, 1000-2000 gr, and > 2000 gr). Group comparisons were performed using ANOVA models. Correlations between continuous variables were estimated using Pearson's coefficient. A two-sided p value < 0.05 was considered for statistical significance. All statistical analyses were performed using SAS Release 9.4 (SAS Institute, Cary, NC, USA).

The analysis of the macronutrients of the milk donated by the mothers of preterm and full-term infants was performed.

The instrument used to evaluate the macronutrients of the donated milk is the MIRIS Human Milk Analyzer, an infrared spectroscopic method that analyzes the composition of the breast milk to determine fat content, proteins, lactose, energy, and dry substance.

We compared the composition data of the milk donated by mothers of preterm and full-term babies from both single and pooled donors.

We continued analyzing the average composition of 300 samples of “preterm” milk and 600 samples of donated milk (300 from a single donor and 300 from a pool). The pooled milk is pasteurized, in contrast to the milk of a single donor which is unpasteurized.

## 5. Results

The HMB of CSS has recruited 659 donors totalling 2236 liters of milk in 7 years.

38 donors (5.7%) are of gestational age <35 weeks. Within this category, 22 donors (57.9%) gave birth prematurely with GA <32 weeks, 11 (28%) with GA of 33 weeks, and 5 (12.8%) with GA of 34 weeks. More precisely, 7 donors (18,4%) are mothers of extremely premature babies with GA ≤ 25 weeks, 1 donor gave birth at 25 weeks, 1 donor at 24 weeks, and 4 donors at 23 weeks and even at an extreme gestational age of 22 weeks. This last woman succeeded at donating about 4 liters of milk within the short stay of 6 days until the inevitable death of the newborn baby. Data include the special donations of a mother of preterm twins (27 weeks of GA and a birth weight of 950 grams and 1100 grams) who devoted 50 liters. 19,9% of the total milk donation was derived from mothers of premature infants with an average of 11732 ml compared to 2936 ml of the term donation.

On the basis of the birth weight, 17 newborns (44,7%) are VLBW with BW <1500 grams, 14 newborns (36,8%) are ELBW with birth weight <1000 gr, and 5 of them (13,1%) weigh less than 500 grams.

In short, the 7 donors giving birth before the 25 weeks gave 100360 ml to the HMB corresponding to 22,5% of the entire “preterm” donation, the category of the 15 mothers of premature GA > 25 wk and < 32 wk donated 254750 ml corresponding to 57.1%, and the last of 16 women with delivery ≥ 32 wk donated 90730 ml which correspond to a percentage of 20,4%.

Considering the birth weight variable, the donors who were mothers of babies with ELBW (<1000 gr) donated 251560 ml to the HMB equal to 56,4% of the total “preterm” donation, the mothers of babies with BW (>1000 gr and <2000gr) devoted 137000 ml equal to 30,7%, and the ones of babies with BW > 2000 donated 38430 ml (13,2%). (Table 1)

DM mean ± standard deviation and ANOVA test according to GA and BW classes are summarized in the tables (Tables 2, 3, and 4).

Regarding the correlation analysis, DM resulted in negative correlation with GA weeks (r=-0.31, p=0.058) and with BW g (r=-0.30, p=0.068), values which are very close to the significance.

In terms of average value, the composition of the “preterm” breast milk is 1.45 gr for proteins, 3.9 g/dl for lipids, 6.48 g/dl for carbohydrates, and a caloric value of 65.7. As widely described, the most variable data relate to lipids having a minimum value of 1.2 g/dl and a maximum of 7.3 g/dl; the ranges of the other macronutrients are (as regards the proteins) the values of 0.7 g/dl and 1.6 g/dl, for carbohydrates 3.2 g/dl and 7.2 g/dl and for calories 35 and 101 Kcal.

The average of the proteins in the milk devoted by individual donors was 1.19 g/dl with a range from 0.5 to 1.97 g/dl. As regards the lipids, the average was 3.95 g/dl with a wide range between a maximum of 12.1 and a minimum of 0.6 g/dl.

The total average, as regards the carbohydrates content, was 6.21g/dl between the maximum and minimum values of 7.48 g/dl and 4.76 g/dl, respectively.

With regard to the energetic value of the donated milk, the average was 64.4 kcal (max 130, min 29).

The nutritional value of the pools is, on average, 1.28 g/dl for proteins, 2.95 g/dl for lipids, 6.87g/dl for carbohydrates, and 58.6 Kcal for energy.

After the pasteurization, we observed a mean reduction of 25% of the protein content and of 29% of the lipid content and a loss of 13% for the energetic value regarding the calories (Table 4); the lactose did not undergo any significant reduction. There is an increase by 18% in the protein component of the preterm milk; if we consider the pooled milk, the gap in carbohydrates is fewer than 6% and only 5% compared to the milk of a single donor. The data on the lipids line up with the milk donated by the single donor who gave birth at term; on the contrary, it grows by 24% compared to the pooled one. The energy value of the preterm milk is 2% higher than the term milk of a single donor and 11% higher than the milk from a pool (Table 5).

## 6. Discussion

In recent years, the feeding of the premature baby has become, rightly, a priority in the assistance of this category of newborns; the breast milk is considered an “essential drug” in particular for VLBW. It is now proven that an incorrect nutrition during the first weeks of life results in significant negative consequences in the short and long terms [8, 9]. The breast milk has the status of a gold standard food and it is recommended by the scientific community (AAP, ESPGHAN) and not only (WHO/ UNICEF) as the first choice for premature infants. This is linked to the numerous benefits of feeding with breast milk, starting from the reduced risk of NEC, sepsis, other comorbidities [18–35] (ROP, BPD), a better enteral tolerance with a faster achievement of the full diet [33], as well as positive effects on neurocognitive and behavioral development, long-term visual acuity, and a reduction of the risk of metabolic and degenerative diseases during the adolescence and the adulthood [9–16]. All this is attributable to the countless biocomponents present in the breast milk. There are benefits linked to the feeding with donated milk, even if they are less specified and demonstrated, particularly in reducing the NEC [18–25]. For this reason, when the mother's milk is not available, the alternative is the human milk donated to the milk banks.

It is estimated that the donated milk involves just 30% of the needs of the premature infants. The milk comes from the generous donors, who are mothers of full-term newborns, who address part of their surplus production to the milk banks.

But do mothers of the premature babies devote their milk to the banks? To what extent can we benefit from this precious food?

Some research [34–36] aimed at quantifying the milk production of the mothers of premature infants gives us some indication on the average production that is about 500-600 ml a day for about 3-4 weeks. But, is it possible to know how many women, who gave birth prematurely, are able to feed their children exclusively with their own milk and also to donate milk to the HMB? In our NICU the enteral feeding of premature infants is activated in the first hours after birth with DM. At the same time, after delivery, mothers are provided with breast pumps for the squeezing of the breasts carried out for the first time within 6 hours from the birth and then every 3 hours in order to obtain an appropriate and early breast stimulation. As soon as the mother's milk becomes available, the bank milk is stopped and replaced by the breast milk characterized by gradual increases as per dedicated protocol.

Since 2010, a HMB has been active in CSS collecting about 2236 liters of milk. 413 liters (19,9%) come from mothers of newborns with GA < 35 weeks. This is the category of donors recruited for the study that corresponds to 5,7% of the total donors. This percentage, although not comparable, can certainly be improved, but the data become more significant if we consider that higher percentages of donation have been observed in the categories with lower GA and BW.

Meanwhile, almost 20% of the milk comes from mothers of premature babies. It means that women who donate “preterm milk” are less numerous but produce more milk and shows the enormous potential of this category of mothers in terms of donation. This result is particularly relevant if we consider that this kind of milk is unique for its biological characteristics, so much that it represents a precious element to safeguard.

With consideration to the birth weight parameter, it was found that VLBW mothers contributed for at least 56% to preterm donation coming from 17 donors (44,7%), while ELBW donors contributed at a rate of 41%, coming from 11 (29%) donors.

Considering the GA variable, about 40% of the average total donation of the mothers of premature babies derives from women who gave birth before the 25 weeks; meanwhile the category of newborns with GA between 25 and 32 weeks contributed at a rate of 46%.

Regarding the correlation analysis, DM was negative in relation to GA weeks (r=-0.31, p=0.058) and to BW g (r=-0.30, p=0.068) achieving values which are very close to the significance.

This is a fact that has never been reported in the literature with the exception of our previous study [37] in which an inverse proportionality between DM and gestational age <29 weeks was outlined.

The comparison between the donor volume averages of the two groups (preterm and full term), corresponding, respectively, to 11732 ml and 2936 ml, is statistically significant (p = 0.007).

However, there are extraordinary examples if we consider the donation of 4 liters done in a few days by a mother who gave birth at a gestational age of 22 weeks and the case of a woman with a twin pregnancy, born at 27 weeks and weighing 950 and 1100 grams. She managed to devote 11% of the entire “preterm” donation. This mother breast-fed her two twins, who grew so well to postpone the weaning period; she devoted more than 50 liters of milk in a period of 6 months (limit specified).

These evidences substantiate the data found in the literature regarding an increase of breastfeeding in the last years, especially in the context of a subgroup of women who historically are less predisposed to breastfeed (mothers of premature babies with a very low weight) [38]. This deals with the unknown reasons that prompt several mothers, despite the shorter duration of their mammary tissue development, to produce so much milk to cover the demands of their children and beyond. A probable correlation is established between the emotional challenges faced by mothers after a premature delivery [39–41]. It is well known that the motivation plays a central role and probably the maternal age of the women recruited should be pointed out: mostly over 35 years of age (about 60%), they should be educated for a greater awareness about the necessity and the importance of the breastfeeding, considering their level of education, which is medium-high. Moreover, it has been proven that the information and the advices given to the mothers of VLBW on the benefits and on the practices of the breastfeeding, do not represent an additional risk factor for the maternal stress but they become a way to make women (who had a premature or difficult delivery) aware and coprotagonists of the care of their children, even when the clinical conditions of the newborns are critical [42, 43].

In our experience, the respect of the protocol regarding the early breast squeezing, within 6 hours after birth and every 3 hours in the following days, is essential for the success of the breastfeeding.

Regarding the composition data, the increase by 18% of the protein component in the milk coming from mothers of premature infants coincides with the literature [44], while the gap of carbohydrates is less significant (5-6%), if we consider the pooled milk, and only 5% if we consider the milk of a single donor; the caloric gap is only 2% higher than the single donor milk and 11% higher in the pooled milk. The data on lipids line up with the single donor term milk, while it becomes more than 24% compared to the pooled one. As reported in the literature, the data on the lipids are the most variable because they depend on the diet and, in the case of donors, also on the time chosen during the day for the donation and on the duration of the expression of the milk that is directly proportional to the lipid secretion. We should bear in mind that the breast milk needs a fortifying powder that compensates for caloric and protein necessities in order to avoid the risk of growth retardation with subsequent negative effects on the development. In our center this process is personalized and executed starting from the milk composition data.

The care of mothers committing to obtain milk during the stay in NICU of their child prematurely born, must be broad with a complete and standardized support [38, 45, 46], in compliance with an extensive and punctual protocol that is of practical and simple use, containing indications of proven efficacy in terms of time, methods, and practices regarding the technical and logistic support on the breastfeeding before with extracted milk and after directly at the breast [47–52].

An element which favours the use of human milk in NICU is the presence in our structure of the Milk Bank with spaces, management, and a dedicated staff that (we believe) has encouraged the production, the maintenance, and the donation. It should be recalled, in this regard, that the presence of a HMB [53, 54] does not interfere with the feeding of premature babies but promotes it. Indeed, the milk banks are a valid system for the collection, the treatment, and the storage of the milk and represent an excellent method to promote the breastfeeding [36]. It is an important commitment at global level because it represents an opportunity for the health and a global intervention on sustainability, ecocompatibility, and fairness.

## 7. Conclusions

The study shows that, even at very low or extreme gestational age or at VLBW and ELBW, there is the possibility of obtaining a suitable milk production from both a quantitative and qualitative point of view. This awareness becomes the fundamental starting point for the triggering, in a standard way, of all those strategies, for the promotion and the support of the breastfeeding in NICU, that have been proven effective.

We emphasize the need of a wider spread of the culture on the maternal breastfeeding for the premature infants but also of the donation, operating on the sense of solidarity and on the generosity of the mothers, as we experienced with the HMB, regardless of their different characteristics and origins. It is important to promote the production and the storage in NICU of the milk thanks to the mothers of premature babies. It is desirable to have a greater distribution of the milk banks in the (not only national) territory, where the donations represent for many newborns an investment and a goal, in terms of well-being.

Finally, an enrichment of the literature on the production data of the preterm milk and the feeding of premature babies shall be advised: it could be the starting point to improve this aspect of the assistance in NICU which is so important to condition the present and the future existence of the delicate population of newborns.

## Figures and Tables

**Table 1:** Total donor milk (ml) according to gestational age and birth weight categories.

**GA (weeks)**	**DM Volume (ml)**	**BW (gr)**	**DM Volume (ml)**
22-25 (N=7)	100360	<1000 (N=11)	251560

26-32 (N=15)	254750	1001-2000(N=18)	137000

33-35 (N=16)	90730	>2000(N=9)	59280

DM: donor milk, GA: gestational age, and BW: birth weight.

**Table 2:** Clinical subjects characteristics.

**Variable**	**Category**	**All Subjects**
n		38

BW		1502.89±684.54

BW_CAT	BW<=1000	11 (28.95)

	1001<=BW<=2000	18 (47.37)

	BW>=2001	9 (23.68)

DM		11732.63±16047.80

GA		29.79±3.89

GA_CAT	22<=GA<=25	7 (18.42)

	26<=GA<=32	15 (39.47)

	33<=GA<=35	16 (42.11)

DM: donor milk, GA: gestational age, and BW: birth weight. Mean ± standard deviation and frequencies and percentages.

**Table 3:** Mean (± standard deviation) of donor milk according to gestational age categories.

**Variable**	**22<=GA<=25**	**26<=GA<=32**	**33<=GA<=35**	**p-value**
n	7	15	16	

DM	14337.14±14142.78	16983.33±21711.71	5670.63±6732.98	0.1299

DM: donor milk, GA: gestational age, and BW: birth weight.

**Table 4:** Mean (± standard deviation) of donor milk according to birth weight categories.

**Variable**	**BW<=1000**	**1001<=BW<=2000**	**BW>=2001**	**p-value**
n	11	18	9	

DM	18323.64±23708.89	11436.11±12848.82	4270.00±3878.54	0.1495

DM: donor milk, GA: gestational age, and BW: birth weight.

**Table 5:** Composition data comparison.

	**“Preterm” milk**	** “Term” milk from a single donor**		**“Term” milk from a pool**	
**Protein (gr/dl)**	1,45	1,19	+18%	1,28	+ 11,73

**Fat (gr/dl)**	3,9	3,95	=	2,95	+ 24,36

**Carbohydrates** **(gr/dl)**	6,48	6,21	+5%	6,87	-6%

**Energy (Kcal)**	65,7	64,4	+2%	58,6	+11%

## Data Availability

The data used to support the findings of this study are available from the corresponding author upon request.

## Abbreviations

HMB: Human Milk Bank
CSS: Casa Sollievo della Sofferenza
VLBW: Birth weight<1500 grams
ELBW: Birth weight<1000 grams
GA: Gestational age
NEC: Necrotizing enterocolitis
DM: Donor milk
BW: Birth weight
ROP: Retinopathy of prematurity
BPD: Bronchopulmonary dysplasia.
